# Gender specific considerations in septorhinoplasty, a retrospective observational study and review of the literature

**DOI:** 10.1016/j.amsu.2022.103810

**Published:** 2022-05-18

**Authors:** S.L. Gillanders, M. Walsh, S. Anderson, S. Abdulrahman

**Affiliations:** Royal College of Surgeon's Ireland, Tallaght University Hospital, Ireland

**Keywords:** Rhinoplasty, Septorhinoplasty, Outcomes, Gender differences

## Abstract

**Introduction:**

In our service experience, we found we had a high proportion of male patients undergoing septorhinoplasty. This encouraged us to research gender specific differences in anatomy, surgical techniques, expectations and outcomes.

**Methods:**

We performed a retrospective chart review of patients who have had rhinoplasty surgery under a single otolaryngology consultant with a special interest in rhinoplasty. Patient information and results of the 10-Item Standardized Cosmesis and Health Nasal Outcomes Survey for Functional and Cosmetic Rhinoplasty pre and post-surgery were collected.

**Results:**

There was no statistically significant difference in the mean pre-operative symptom (29.31 vs 32.29 p = 0.559), change in symptom (23.25 vs 24.14 p = 0.827) or satisfaction scores (8.69 vs 7.29 p = 0.089) between male and female patients. A discussion on gender specific anatomical features and deformities is presented.

**Conclusion:**

All patients reported improved symptoms and high levels of satisfaction. Careful patient counselling and patient-specific surgical planning help to achieve optimal outcomes.

## Highlights

• Traumatic nasal bone injury is a common cause of cosmetic and functional issues seen in the Otolaryngology outpatients.
• Limited research exists on male rhinoplasty despite its growing popularity.
• Knowledge of individual anatomical variations and surgical technique is key to providing personalised patient care.
• Careful patient counselling and patient specific surgical planning help to achieve optimal outcomes.

## 1 Introduction

Careful patient selection is key for ensuring cosmetic and functional satisfaction in the rhinoplasty patient cohort. For otolaryngologists functional outcomes were historically paramount however, cosmetic adjustments are becoming standard in most rhinology practices [1]. With this evolution in practice and patient priorities, the expectations of our patients have also changed.

Managing patient expectations has been part of every surgeons practice however, lengthy interactions and follow-up with unhappy manipulative, deceptive or unrealistic patients are seldom encountered in general otolaryngology practice. Cosmetic surgery opens a different element of subjective findings not easily measurable by functional outcomes or data.

Cosmetic rhinoplasty surgeons have long advocated patient screening to identify specific personality traits, anxiety and body dysmorphic disorders with new referrals. Careful patient selection and communication is key to preventing patient dissatisfaction cumulating in hours of unhappy consultation, poor surgical outcomes and even medicolegal concerns. The pneumonic “SIMON” (single, immature, male, overly expectant, narcissistic) was coined by Gorney [2] to warn against operating on this particularly difficult patient.

On reviewing our service experience, we found we had a high proportion of male patients in our cohort. This encouraged us to research gender specific differences in anatomy, surgical techniques, expectations and outcomes. The aim of this study is to identify these gender specific considerations, add to the sparse literature available on male aesthetic considerations and encourage readers to utilise this in their own practice.

## 2 Materials and methods

We performed a retrospective paper and electronic chart review of patients who have had rhinoplasty surgery under a single otolaryngology consultant with a special interest in rhinoplasty. All patients over the age of 18 operated between January 2018 and October 2020 were included. This ensured all patients had attended a 6-month review following their surgery however, the impact of covid-19 during this period has limited our ability to perform this in a physical clinical setting.

Digital photography was performed pre and post-surgery in all patients for recordkeeping, planning, and comparison. Patient demographics, GP referrals, patient concerns/goals, treatment details, and outcomes were collected including results of the 10-Item Standardized Cosmesis and Health Nasal Outcomes Survey [3] for Functional and Cosmetic Rhinoplasty pre and post-surgery. Patient data and outcomes were compiled and analysed in terms of mean and standard deviations. Measures of central tendency and variance were calculated. The independent *t*-test was applied with a confidence interval of 95%. P < 0.05 was considered statistically significant. Pearson Chi-square was used to analyse categorical variables. Data analysis was done using IBM Statistical Package for Social Sciences (SPSS) statistical software, version 25. Work was reported in line with STROCSS criteria [4] and registered with Research Registry 7908.

## 3 Results and analysis

A total of 25 patients were identified. 5 patients were excluded due to language barriers and consent reasons. Of the 20 patients included 13 were male and 7 female. 16 cases were as a result of trauma (5 sports related, 4 assault, 7 accident) and 4 base line aesthetic complaints. All baseline aesthetic complaints were female patients (Pearson Chi-square 9.286 p = 0.002) and all sports related injuries were male patients (Pearson Chi-square 3.59 p = 0.058). Nasal obstruction was reported in all but one case. There was no statistically significant difference in the mean pre-operative symptom scores (29.31 vs 32.29 p = 0.559), change in symptom scores (23.25 vs 24.14 p = 0.827) or satisfaction score (8.69 vs 7.29 p = 0.089) between male and female patients.

In addition, no statistically significant difference was found between patients based on reason for deformity (trauma versus baseline aesthetic concern) or whether GP referral matched patient concern.

The type of surgery performed did appear to have some variation between genders. In both groups septoplasty was performed in all bar one case and dorsal hump correction (including all techniques) was evenly required. However, osteotomies were required more often in male patients 84.6% versus female patients 42.9%. (Pearson Chi-square 3.778 p = 0.052) and tip work was relatively more common in female patients 57.1% versus 38.5% however not significantly so. Sutures tended to be favoured in female patients and grafts in male patients.

## 4 Discussion

### 4.1 Anatomical differences and surgical technique

Anatomical differences between the traditional male and female nose are important to consider when planning an individualised surgical procedure. For Caucasian patients the suggested optimal nasofrontal angle for male patients is 129° compared to 144.5° in female patients. The radix is ideally located at the upper eyelid crease in men slightly higher than in women at just above the level of the pupil. In addition, projection is greater in men with an average of 34 mm versus 29.6 mm in females [5]. Dorsal aesthetic lines ideally are straight in the masculine nose compared to concave in the feminine nose [6]. Overall, the male nose is often larger, with thicker skin and a wider base. The three most common baseline aesthetic issues in the male nose are prominent dorsal humps, wide nasal bones, and poor definition of the tip. In our experience most male rhinoplasty patients had deviated nasal bones from trauma or sporting injuries and nasal obstruction was the greatest concern.

Unfortunately there is limited literature on male rhinoplasty aesthetic goals, as historically this was a predominantly female market. What would routinely be considered aesthetic goals in the female nose would not be acceptable in a cisgender male patient. Excessive reduction of the dorsum, narrowing of the dorsal aesthetic lines, and refinement of the nasal tip can lead to over feminisation of the male nose. Conversely, it is useful to note that these techniques may be employed in gender-reassignment/gender-confirming surgery for this very outcome.

### 4.2 Expectations and outcomes

Within the literature, the male aesthetic patient in general is known to have poorer satisfaction scores [7]. In keeping with this, several papers have reported greater patient satisfaction in female rhinoplasty patients over male rhinoplasty patients [8,9]. In one large study, in-depth analysis of reasons for dissatisfaction were similar between the two genders, with under correction of original deformity most often sited. However, patients with a deviated nose or previous injury were the most likely to be satisfied with their results despite the technical challenges surrounding this difficult surgery. Interestingly, many authors report their male patients were more vague in both describing pre-operative goals and post-operative concerns [10]. Despite being more dissatisfied than their female counterparts, male patients were unable to specify their functional or cosmetic concern. Instead comments of emotion and regret were provided as explanation [8]. In our study we did not find a statistical difference between male and female patients, this may be explained as the majority of cases were traumatic injuries with a heavily weighted functional concern. From experience this would be in keeping with most Irish otolaryngology surgeons’ practice.

### 4.3 Limitations of paper

This was a small patient cohort from a single surgeons experience over a short time frame. Service restrictions and resource allocations within Ireland means functional septo-rhinoplasty is not easily accessible in the public sector and so large numbers for inclusion would be difficult to achieve.

The covid-19 impact on access to surgery in the public sector has been mentioned previously in the paper. In addition to this, the use of face-masks and social restrictions of lock-down may have helped camouflage the impact of a cosmetic deformity and reduced referral for correction.

In addition, measurement of patient satisfaction, cosmetic and functional outcomes is difficult [11]. Several assessment tools have been created for individual outcomes aesthetic or function but few including both. Surgeon and patient opinions on cosmetic outcomes can often disagree [12] and even objective measures of success may not accurately reflect patient satisfaction [13]. We chose to use SCHNOS as our assessment tool because this focused on patient reported outcomes and included both aesthetic and functional elements. However, one concern for a surgeon initiated survey like SCHNOS is bias, as patients may be reluctant to express their true feelings of dissatisfaction towards the surgeon [8]. This study would be interesting to compare national figures and also surgeries performed in the private sector to increase recruitment numbers and diversify the patient cohort.

### 4.4 Future implications

The complex demands of rhinoplasty patients on Otolaryngologists will continue to increase in our clinical practice. The rising concern on combined functional and cosmetic outcomes, a holistic patient care approach and the expanding subset of facial plastics within the rhinologists surgical repertoire are all areas in need of development. The separation or neglect of one aspect of cosmetic or functional outcomes is no longer acceptable. This author feels that further research and effort should be applied to aid in correct patient selection, individualised patient surgical planning, and application of simple, accurate evaluation methods pre and post-surgery to achieve optimal outcomes including patient satisfaction.

## 5 Conclusion

Overall, both male and female patients within this group reported improved symptoms and high levels of satisfaction. There was no significant difference in any outcome analysed. Careful patient counselling and patient specific surgical planning help to achieve optimal outcomes.

## Ethical standards

The authors assert that all procedures contributing to this work comply with the ethical standards of the relevant national and institutional guidelines on human experimentation and with the Helsinki Declaration of 1975, as revised in 2008.

## Provenance and peer review

Not commissioned, externally peer-reviewed.

## Ethical approval

SJH/TUH Research Ethics Committee Secretariat email: researchethics@tuh.ie REC: 2021-04 Chairman's Action (16).

## Sources of funding

Nil.

## Author contribution

SL Gillanders, data collection, first/corresponding author.

M Walsh, data collection, editor.

S Anderson, statistics, editor.

S Abdulrahman, senior author, editor.

## Registration of research studies

1. Name of the registry: Research Registry.
2. Unique Identifying number or registration ID: 7908.
3. Hyperlink to your specific registration (must be publicly accessible and will be checked): https://www.researchregistry.com/browse-the-registry#home/registrationdetails/627e8f8798f9f100217da784/.

## Guarantor

SL Gillanders.

## Consent

SJH/TUH Research Ethics Committee Secretariat email: researchethics@tuh.ie REC: 2021-04 Chairman's Action (16).

## Declaration of competing interest

Nil.

## References

[1] Burns P. (2008). Otorhinolaryngologists' interest in facial plastic surgery: a survey in the United Kingdom and Ireland. J. Laryngol. Otol..

[2] Gorney M., Martello J. (1999). Patient selection criteria. Clin. Plast. Surg..

[3] Moubayed S.P. (2018). The 10-item standardized Cosmesis and Health nasal outcomes survey (SCHNOS) for functional and cosmetic rhinoplasty. JAMA Facial Plast. Surg..

[4] Mathew G., Agha R. (2021). STROCSS 2021: strengthening the reporting of cohort, cross-sectional and case-control studies in surgery. Int. J. Surg..

[5] Springer I.N. (2008). Gender and nasal shape: measures for rhinoplasty. Plast. Reconstr. Surg..

[6] Berli J.U., Loyo M. (2019). Gender-confirming rhinoplasty. Facial Plast. Surg. Clin. North Am..

[7] Wright M.R. (1987). The male aesthetic patient. Arch. Otolaryngol. Head Neck Surg..

[8] Khansa I., Khansa L., Pearson G.D. (2016). Patient satisfaction after rhinoplasty: a social media analysis. Aesthetic Surg. J..

[9] Khan N. (2019). Satisfaction in patients after rhinoplasty using the rhinoplasty outcome evaluation questionnaire. Cureus.

[10] Rohrich R.J., Janis J.E., Kenkel J.M. (2003). Male rhinoplasty. Plast. Reconstr. Surg..

[11] Soni K. (2020). Post-rhinoplasty outcomes in an Indian population assessed using the FACE-Q appraisal scales: a prospective observational study. J. Laryngol. Otol..

[12] Shipchandler T.Z. (2013). Aesthetic analysis in rhinoplasty: surgeon vs. patient perspectives: a prospective, blinded study. Am. J. Otolaryngol..

[13] Spörri S. (2004). Objective assessment of tip projection and the nasolabial angle in rhinoplasty. Arch. Facial Plast. Surg..

